# Nodular Eruptions as a Rare Complication of Botulinum Neurotoxin Type-A: Case Series and Review of Literature

**DOI:** 10.7759/cureus.10175

**Published:** 2020-08-31

**Authors:** Mohammed Anabtawi, James Wege, Hanya Mahmood, Bahaa Alsaeed Dareen Amro, Alan Patterson

**Affiliations:** 1 Oral and Maxillofacial Surgery, Chesterfield Royal Hospital, Chesterfield, GBR; 2 Oral and Maxillofacial Surgery, Rotherham General Hospital NHS Foundation Trust, Rotherham, GBR; 3 Oral and Maxillofacial Surgery, Rotherham National Health Service Foundation Trust, Rotherham, GBR; 4 Oral Surgery, University of Sheffield, Sheffield, GBR; 5 Dentistry, Private Clinic, Hebron, PSE

**Keywords:** botulinum neurotoxin type-a, granuloma, sarcoid, lump, nodules, botox

## Abstract

Nodular eruption after botulinum neurotoxin type-A (BoNT-A) treatment is exceedingly rare, and the pathogenesis is poorly understood. This case series reports three patients that developed nodular eruptions following administration of Botox® (onabotulinum neurotoxin type A (ONA) injections). These patients had undergone multiple treatments before and after development of the eruptions which were uneventful. In addition to this, we have reviewed the published literature regarding this condition and have compared and contrasted the similarities and differences with regards to the clinical presentation and treatment with our patient cohort. This case series aims to raise awareness of this rare condition, its importance in relation to patient consent and provides a simplified management approach based on our experience. Further evaluation is needed to determine treatment consensus but conducting such research may prove to be challenging due to this condition being an infrequent encounter.

## Introduction

Treatment with botulinum neurotoxin type A (BoNT-A) injections is a popular and well-established treatment option for management of various medical conditions and cosmetic ailments. Common side effects include pain, itching, erythema and bruising. Rarer complications include squinting, ptosis, and difficulty swallowing and breathing, which are mainly caused by diffusion of the toxins to nearby structures. These are usually temporary and self-limiting. More serious side effects are likely to result from therapeutic doses in contrast to cosmetic ones [1].

Skin nodules and lumps are rarely seen as a side effect of botulinum toxin treatment, with only five reported cases in the literature. These nodules (granulomas) can cause significant distress for both patients and clinicians, especially as there is no known cause and a lack of consensus on management.

This case series illustrates three patients who developed nodular eruptions shortly after administration of onabotulinum neurotoxin type A (ONA) injections. We have described the clinical presentation and management of this rare side effect, in addition to reviewing the existing literature for reported cases of this rare condition to allow comparison of similarities and differences.

A retrospective review of three patients that developed nodular eruptions following administration of ONA (Botox®, Allergan, Irvine, CA, US) was undertaken. All patients had ONA for cosmetic purposes and treatment was provided by two different clinicians (August and September 2017) at the same clinic. In all cases, the Botox® ampoules were from the same batch.

The clinic followed a standard protocol in Botox® reconstitution and surgical site preparation. Botox® was prepared as per the standard technique advised by the manufacturer. The clinic has been using the same brand and formulation of normal saline and needles for all treatments conducted over the last five years. The patients were not wearing make-up on the day of treatment.

In addition, electronic database searches in EMBASE and MEDLINE were conducted to retrieve all papers related to this subject (up to March 2020) with assistance from a medical information specialist at Rotherham General Hospital, UK. The principal inclusion criteria were studies that investigated the presentation and/or treatment of nodules following injection of BoNT-A.

IRB is not applicable to this case series and review of literature.

## Case presentation

Case 1

**Figure 1:**
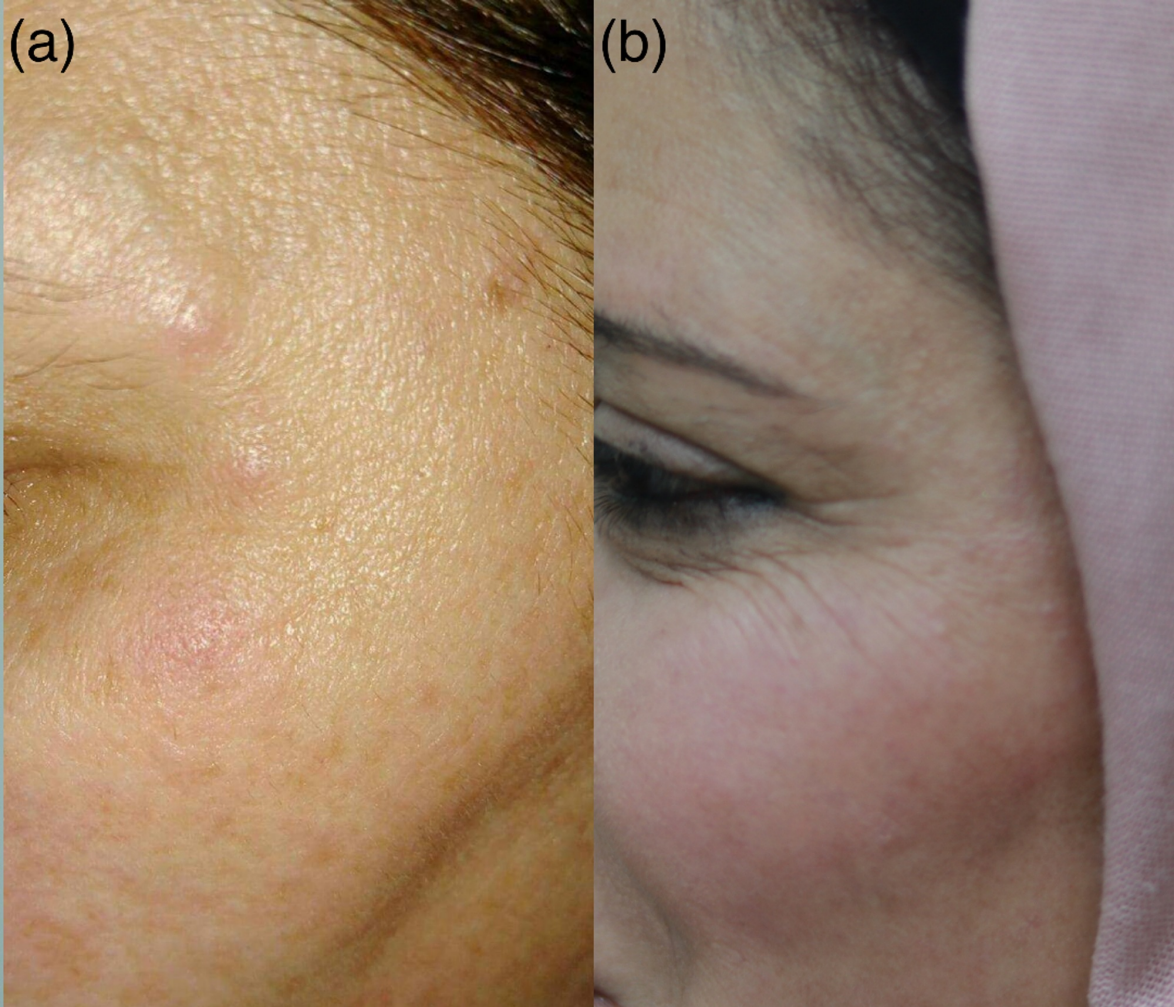
Case 1 A female patient who was treated with botulinum neurotoxin type-A (BoNT-A) to the crow’s feet area, presented after a week with those nodules at the site of injections (a), and the same patient at 32 months follow-up showing complete healing without scarring (b).

A healthy 45-year-old female underwent treatment with Botox® (ONA) in August 2017 to the crow’s feet area for cosmetic reasons. It was her first-time having botulinum toxin injections (Figure 1). After one week, the patient presented with multiple firm well-defined itchy red swellings at the injection sites. She was treated with a one-month course of Doxycycline 100mg ODS, 20mg Prednisolone ODS for seven days then 10mg for seven days, with a two-week course of topical cream containing both Gentamicin sulphate 0.16% and Prednisolone 0.5%. After two weeks, the redness and itching disappeared completely, whilst the nodules, which had a normal skin appearance took six months to resolve completely.

Case 2

**Figure 2:**
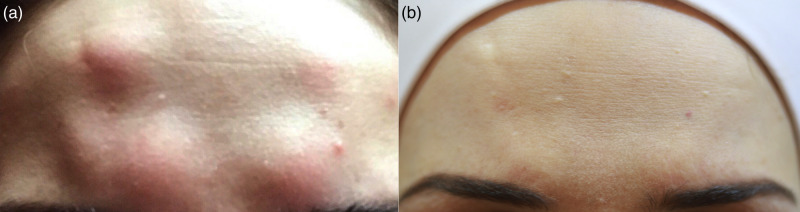
Case 2 A female patient who was treated with botulinum neurotoxin type-A (BoNT-A) to the crow’s feet, forehead and glabella areas, presented after two days with those nodules at the site of all injections (a), and the same patient at 32 months follow-up showing complete healing without scarring (b).

A healthy 34-year-old female was treated with Botox® (ONA) in September 2017 (Figure 2). The areas treated were the glabella, forehead and crow’s feet bilaterally for cosmetic reasons. This was not her first course of botulinum toxin injections; previous treatments were uneventful. Two days after administration, she presented with similar multiple firm well-defined itchy red swellings at all sites of injections. This was treated with a one-week course of topical cream containing both Gentamicin sulphate 0.16% and Prednisolone 0.5%. Like the first patient, after two weeks, the redness and itching disappeared, whilst swelling took six months to resolve completely.

Case 3

**Figure 3:**
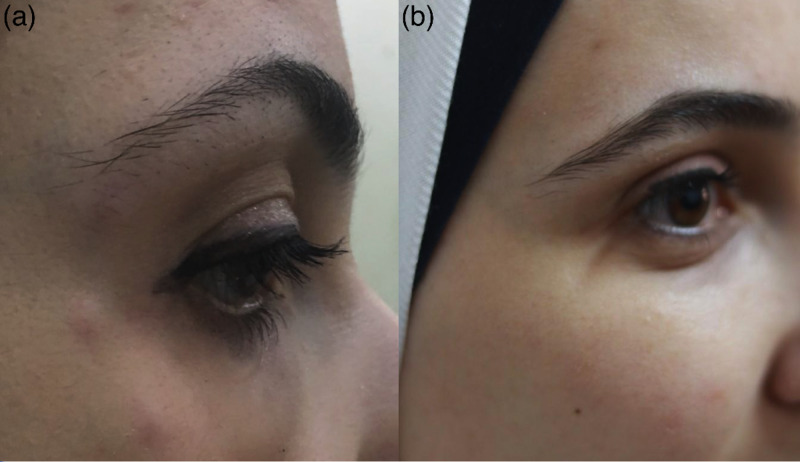
Case 3 A female patient who was treated with botulinum neurotoxin type-A (BoNT-A) to the crow’s feet area bilaterally and one point of injection to the left corrugator muscle, presented after three weeks with those nodules at the site of injections in the crow’s feet area bilaterally, but not at the site of corrugator injection (a), and the same patient at 32 months follow-up showing complete healing without scarring (b).

A healthy 27-year-old female treated with Botox® (ONA) in September 2017 (Figure 3). The areas treated were the crow’s feet bilaterally and one point of injection to the left corrugator muscle, again for cosmetic reasons. This was not her first course of botulinum neurotoxin injections. Three weeks later, the patient presented with similar multiple firm well-defined itchy red swellings at the sites of crow’s feet injections but not to the glabella area. It was treated with a one-week course of topical cream containing both Gentamicin sulphate 0.16% and Prednisolone 0.5%. Like the other two cases, after two weeks, the redness and itching disappeared, whilst swelling took six months to resolve completely.

All three patients went on to have further BoNT-A treatment without any further complications. As can be seen above, all three patients had similar clinical presentations, firm itchy red nodules at the sites of botulinum toxin injection, with barely any variations in the clinical findings, suggesting similar aetiology of the nodules.

After discussion with the patients about the possible aetiology of the lumps, biopsies and a combination of topical and systemic steroids/antibiotics were offered. All three patients opted against a biopsy unless the lesions persisted, as a biopsy was unlikely to affect the initial management. Two of the patients opted not to start with systemic steroids as they were happy to see if the lumps would respond to the topical treatment. All three patients were reviewed regularly until the time of submitting this paper (32 months in total). There were no signs of recurrence when treated with further BoNT-A injections, and there were no signs or symptoms of any other systemic diseases.

## Discussion

With the increased use of BoNT-A, it is likely that clinicians may encounter some of the rare side effects of this treatment, including nodular eruptions. This side effect may cause a lot of distress to the patient and their treating clinician.

On review of the existing literature, there were only five reported cases of nodular eruptions following BoNT-A administration (Table 1). All patients were females and aged between 42 and 57 years old (mean 48.8 years). Medically two patients were diagnosed with sarcoidosis [2,3], another patient had hypertension [4], and the other two patients were fit and well. Across all the studies, the lesions presented at the same locations of the BoNT-A injections, and they appeared between two days and six years after treatment. All patients had a biopsy to confirm diagnosis (Table 1). The histopathological findings were: suppurative granuloma, sarcoid-like granuloma and foreign-body granuloma. Two studies showed an association between nodular eruption and systemic sarcoidosis. Assmann et al. [2] found sarcoid-like granulomas in a patient with systemic sarcoidosis occurred at the site of injection six years after the BoNT-A treatment, whilst Herbert et al. [3] reported sarcoid-like granulomas four years before the diagnosis of sarcoidosis, despite repeated negative results of the same tests at the time of presentation and during the treatment course. Meanwhile, Ahbib et al. [5] reported a case of sarcoid-like granuloma without foreign body involvement at the site of Botox® injections in a patient who had no signs of systemic sarcoidosis. Thanasarnaksorn et al. [4] reported nodular eruption with histopathological findings of suppurative granuloma, which yielded negative results for all organisms on histochemical staining and tissue culture.

**Table 1:** Summary of the cases found in the literature review.

Paper	Treatment indication	Brand used	Past/future treatments	Presentation	Treatment	Treatment length	Time for resolution	Biopsy results	Follow up
Assmann et al. (2013) [2]	Cosmetic	Dysport® (Ipsen Pharma GmbH, Ettlingen, Germany)	Previous injections not documented. No future treatments	6 years after injections. Painless, non-erythematous, symmetrical nodules.	Oral prednisolone 50mg.	4 weeks	4 weeks full resolution	Cutaneous sarcoidal granuloma	Not reported
Herbert et al.(2015) [3]	Cosmetic	Dysport®, (Ipsen Pharma GmbH, Ettlingen, Germany)	Multiple previous treatment. No future treatments.	2 weeks after injection. Painless, erythematous symmetrical nodules.	No success with 5-fluoruoracil and Triamcinolone acetonide intralesional injections, Chloroquine, 120mg oral prednisolone and Azathioprine. Success with Azithromycin for one month.	4 years	4 years 50% reduction in size after one month of Azithromycin	Sarcoidal granuloma	Not reported
Thanasarnaksorn et al. (2019) [4]	Cosmetic	Neuronox® (Medy-Tox Inc, South Korea)	No previous treatment Future treatment not mentioned	4 days after injection. Painful, tender, red nodules.	Levofloxacin 500mg. Clarithromycin 500mg.	6 months	6 weeks – 6 months Full resolution	Suppurative granuloma	Not reported
Ahbib et al. (2006) [5]	Cosmetic	Botox® (Allergan, Irvine, CA, US)	No past/future treatments	3 weeks after injection. Firm non-erythematous, painless fixed nodules and a single red papule.	Triamcinolone acetonide injections. Oral prednisolone 16mg.	4 weeks	6 weeks Full resolution	Sarcoidal granuloma	6 months
Yun et al. (2013) [6]	Cosmetic and masseteric hypertrophy	Botox® (Allergan, Irvine, CA, US) or Botulax® (Hugel Inc, Chuncheon-Si, Gangwon-do, Korea)	Neither documented	2 days after injection. Painful, tender, itchy swellings.	Triamcinolone acetonide intralesional injections. Ciprofloxacin 1000mg. Oral prednisolone 8mg. Oral tranilast 300 mg. Hydroxychloroquine 200 mg. Topical tacrolimus ointment (0.1%).	8 weeks	8 weeks Symptoms subsided and lesion diminished significantly	Foreign body granuloma	Not reported

In our presented case series, nodular eruptions developed between two days and three weeks after administration of Botox®; a timeframe which generally matched what is reported in the literature. The exception being Assmann et al. [2] who reported a case that presented with nodules six years after administration (this was associated with systemic sarcoidosis). Some of the clinical photographs and descriptions found in the case reports appear to match the presentation of the nodules in our cases.

Although two of the studies showed an association between nodular eruption and systemic sarcoidosis [2,3], none of our cases had any history or symptoms of this at presentation, and were reviewed up to 32 months later to ensure this. Nevertheless, granulomatous eruption of cicatricial sarcoidosis at site of scars, intramuscular injections, intradermal application of hyaluronic acid, tattoos, vein puncture, and healed herpes zoster, has been reported to happen anytime from six months to 59 years [7]. Therefore, a long follow-up may be needed to rule it out.

The studies investigated the possible causes of such lesions. A common hypothesis in the literature is that a crystalline preparation of BoNT-A or proteinous components of BoNT-A could elicit a foreign body reaction leading to granuloma formation [2,6]. This is supported by Yun et al. [6] who discussed the foreign-body reaction as a causative factor, which could be due to the proteinous component of BoNT-A product. This can be either the BoNT-A itself or the human serum albumin (HSA) component in the BoNT-A vial. As BoNT-A is a xenogenic protein of bacterial toxin, it may generate type 4 hypersensitivity reaction forming granuloma, while HSA was reported to induce granulomas in mice [8]. The combination of those two components or complexing proteins within the product might have played a certain role in developing granulomas [9]. It is difficult to be certain which of the two products (BoNT-A or HSA) is responsible for this kind of reaction without testing them independently on the same subject.

Ahbib et al. [5] reproduced sarcoid granulomas after Botox® injection in the forearm, but they couldn’t reproduce it with saline injections. This led them to think that this was a local sarcoidosis reaction by antigenic stimulation, like a Kveim reaction, rather than scar reactivation sarcoidosis. Triamcinolone resulted in rapid regression of these nodules.

Despite the negative culture and polymerase chain reaction (PCR) results in the case they have presented, Thanasarnaksorn et al. [4] suggested the role of non-tuberculous mycobacteria in the formation of such nodules. Other possibilities that have been suggested include preparation lubricant, diluent or components of the needle itself.

Due to the small number of reported cases, it is difficult to ascertain the exact aetiology of this rare presentation. The picture is further clouded by the fact that the patients in our cohort did not proceed to have this reaction in previous or future treatments with ONA. This raises a few scientific questions as to the possibility of why these three patients had such similar reactions in a small-time frame, with both host and drug factors needing exploration. One common factor in all three of our patients is the drug used. The manufacturer preparation process or storage of the toxin in certain batches cannot be excluded as a cause.

In the studies reviewed, there was a variation in the duration and type of treatment provided. The treatment duration varied widely between the studies and ranged between four weeks and four years. Different combinations of treatments were used successfully to treat the condition, which included oral/intralesional steroids, antibiotics, oral tranilast, hydroxychloroquine and topical tacrolimus (Table 1). One patient with lesions who was later diagnosed with systemic sarcoidosis did not respond to various therapies including trials of intralesional injections of 5-fluoruoracil and triamcinolone, and oral prednisolone, azathioprine and chloroquine over a period of four years. However, the lesions did respond to a one-month course of azithromycin which reduced the size of the lesions by 50% [3]. The remaining four patients healed completely without scarring within four weeks to six months of presentation.

The rationale behind the use of antibiotics in the treatment of sarcoid granulomas came after a randomised placebo-controlled single-masked trial on 30 patients with symptomatic chronic cutaneous sarcoidosis lesions, conducted by Drake et al. [10]. This study found that antimicrobial therapy (oral concomitant levofloxacin, ethambutol, azithromycin, and rifampin (CLEAR)) causes significant reduction of lesion size and associated symptoms. The study utilised Transcriptome analysis of sarcoidosis CD4+ T cells which showed reversal of pathways associated with disease severity and enhanced T-cell function following T-cell receptor stimulation.

There was some variation in the treatment of our three cases due to different practitioners treating each case and due to patient wishes. Whilst two of the patients declined systemic steroids to treat the lesions and opted to have the topical treatment only, all three cases resolved completely in similar time frames: two weeks for redness and itching to resolve, and six months for complete resolution of the swelling without scarring. This could suggest that the systemic steroids given did not provide any further benefit or that the condition is self-limiting and does not require any treatment.

The length of treatment in the literature and in our cases varied substantially. Our treatment protocol was simple and shorter in duration compared to the previously published cases and resulted in full resolution and no scarring (Tables 1, 2). Again, this raises the question of whether this condition is self-limiting. It is worth mentioning that in our cohort, the patient’s acceptance and satisfaction with the treatment was much improved after two weeks, when itching and redness disappeared, showing that these symptoms were the most concerning for the patients.

**Table 2:** Summary of the features related to the three cases that we treated for BoNT-A-related nodular eruptions. BoNT-A: Botulinum neurotoxin type-A

Case	Treatment indication	Brand used	Past/future treatments	Presentation	Treatment	Treatment length	Time for resolution	Follow up
1	Cosmetic	Botox® (Allergan, Irvine, CA, US)	No past treatments. Future treatments uneventful.	1 week after injections: multiple firm, well-defined, itchy, red swellings.	Topical cream of Gentamicin sulphate 0.16% and Prednisolone 0.5% Doxycycline 100mg Prednisolone 20mg, then 10mg	2 weeks	6 months Full resolution	32 Months
2	Cosmetic	Botox® (Allergan, Irvine, CA, US)	Uneventful past and future treatments	2 days after injections: multiple firm, well-defined, itchy, red swellings.	Topical cream of Gentamicin sulphate 0.16% and Prednisolone 0.5%	1 week	6 months Full resolution	31 Months
3	Cosmetic	Botox® (Allergan, Irvine, CA, US)	Uneventful past and future treatments	3 weeks after injections. multiple firm, well-defined, itchy, red swellings.	Topical cream of Gentamicin sulphate 0.16% and Prednisolone 0.5%	1 week	6 months Full resolution	31 Months

## Conclusions

In summary, the three cases in our cohort demonstrate a relatively similar presentation to other reported cases in the literature. Whilst various treatments have been tried in the past, in our experience of managing this rare condition, we suggest starting with combined topical steroids and antibiotic creams/ointments. If the lumps are not responding to this topical regime, or the patient is showing signs or symptoms of systemic sarcoidosis, we recommend a biopsy from the most diagnostically representative lesion, blood tests and a lung CT-scan to rule out systemic sarcoidosis. In addition to this, we recommend trialling a course of oral antibiotic such as azithromycin or levofloxacin. There should also be regular long-term follow-up to ensure that there is no delayed presentation of sarcoidosis. Whilst the management described in our cohort has been effective and provided complete resolution in all three of our patients, further evaluation and research is necessary to determine consensus on management. However, we recognise that due to the rarity of this condition this may prove to be difficult to undertake. It is acknowledged that the prevalence of such lesions is exceedingly rare, however in the current light of patient consent, it is increasingly important to ensure that all possible risks are appropriately discussed in advance. This will also help manage patient expectations and minimise the risk of litigation particularly in relation to elective cosmetic procedures.
